# Supplementary material for: Identification of TMEM206 proteins as pore of PAORAC/ASOR acid-sensitive chloride channels
Source: eLife. 2019 Jul 18;8:e49187. doi: 10.7554/eLife.49187 (PMC6663466; doi:10.7554/eLife.49187)
Supplement: Transparent reporting form [file elife-49187-transrepform.pdf]

eLIFE
1st Floor
24 Hills Road
Cambridge CB2 1JP, UK
P 01223 855340
W elifesciences.org
T @elife

# *eLife's* transparent reporting form

We encourage authors to provide detailed information *within their submission* to facilitate the interpretation and replication of experiments. Authors can upload supporting documentation to indicate the use of appropriate reporting guidelines for health-related research (see EQUATOR Network), life science research (see the BioSharing Information Resource), or the ARRIVE guidelines for reporting work involving animal research. Where applicable, authors should refer to any relevant reporting standards documents in this form.

If you have any questions, please consult our Journal Policies and/or contact us: editorial@elifesciences.org.

**Sample-size estimation**

- You should state whether an appropriate sample size was computed when the study was being designed
- You should state the statistical method of sample size computation and any required assumptions
- If no explicit power analysis was used, you should describe how you decided what sample (replicate) size (number) to use

Please outline where this information can be found within the submission (e.g., sections or figure legends), or explain why this information doesn't apply to your submission:

**We did not use power analysis to determine sample sizes. We decided on an appropriate sample size by roughly estimating the variability of our recordings, and comparing it to on our own experience and published reports of similar assays. We stuck with predetermined sample sizes.**

**Replicates**

- You should report how often each experiment was performed
- You should include a definition of biological versus technical replication
- The data obtained should be provided and sufficient information should be provided to indicate the number of independent biological and/or technical replicates
- If you encountered any outliers, you should describe how these were handled
- Criteria for exclusion/inclusion of data should be clearly stated
- High-throughput sequence data should be uploaded before submission, with a private link for reviewers provided (these are available from both GEO and ArrayExpress)

Please outline where this information can be found within the submission (e.g., sections or figure legends), or explain why this information doesn't apply to your submission:

**Every dataset on which we performed statistical analysis is from 2-3 independent days/transfections, and we generally only report data that was replicated on at least 2 different days/transfections. For electrophysiological recordings, experiments had to be excluded from analysis if the patch was lost during the experiment (sudden appearance of leak currents), or if series resistance increased above 8 MOhm. We state this in the methods section. In electrophysiological experiments, we mainly focused on parameters obtained from the same cell (e.g. differences in reversal potentials, currents before and after MTSES application). These parameters are unlikely to be affected by day-to-day variation introduced by factors such as transfection efficiency.**

eLife Sciences Publications, Ltd is a limited liability non-profit non-stock corporation incorporated in the State of Delaware, USA, with company number 5030732, and is registered in the UK with company number FC030576 and branch number BR015634 at the address 1st Floor, 24 Hills Road, Cambridge CB2 1JP | August 2014

**Statistical reporting**

- Statistical analysis methods should be described and justified
- Raw data should be presented in figures whenever informative to do so (typically when N per group is less than 10)
- For each experiment, you should identify the statistical tests used, exact values of N, definitions of center, methods of multiple test correction, and dispersion and precision measures (e.g., mean, median, SD, SEM, confidence intervals; and, for the major substantive results, a measure of effect size (e.g., Pearson's r, Cohen's d)
- Report exact p-values wherever possible alongside the summary statistics and 95% confidence intervals. These should be reported for all key questions and not only when the p-value is less than 0.05.

Please outline where this information can be found within the submission (e.g., sections or figure legends), or explain why this information doesn't apply to your submission:

**For maximal transparency, we present the data as scatter plots with individual measurements visible, where this is informative. In all other cases, we report preferentially SD, or SEM. The meaning of the bars/symbols, error ranges, and N are explained in the figure legends. Statistical tests performed, and ranges of resulting P-values are reported directly in the respective figure legends.**

(For large datasets, or papers with a very large number of statistical tests, you may upload a single table file with tests, Ns, etc., with reference to sections in the manuscript.)

**Group allocation**

- Indicate how samples were allocated into experimental groups (in the case of clinical studies, please specify allocation to treatment method); if randomization was used, please also state if restricted randomization was applied
- Indicate if masking was used during group allocation, data collection and/or data analysis

Please outline where this information can be found within the submission (e.g., sections or figure legends), or explain why this information doesn't apply to your submission:

**It is clearly stated in all cases which condition (channel construct, mutant, wild-type, not transfected control) is shown in the respective figures. Appropriate controls (wild-type, not transfected) were independently performed for each individual group of experiments (e.g. within one figure panel). We did not apply randomization or masking.**

**Additional data files ("source data")**

- We encourage you to upload relevant additional data files, such as numerical data that are represented as a graph in a figure, or as a summary table
- Where provided, these should be in the most useful format, and they can be uploaded as "Source data" files linked to a main figure or table
- Include model definition files including the full list of parameters used
- Include code used for data analysis (e.g., R, MatLab)
- Avoid stating that data files are "available upon request"

Please indicate the figures or tables for which source data files have been provided:

**Not applicable**

eLife Sciences Publications, Ltd is a limited liability non-profit non-stock corporation incorporated in the State of Delaware, USA, with company number 5030732, and is registered in the UK with company number FC030576 and branch number BR015634 at the address 1st Floor, 24 Hills Road, Cambridge CB2 1JP | August 2014
